# Photobiomodulation improves functional recovery after mild traumatic brain injury

**DOI:** 10.1002/btm2.10727

**Published:** 2024-10-11

**Authors:** Andrew R. Stevens, Mohammed Hadis, Abhinav Thareja, Freya G. Anderson, Michael R. Milward, Valentina Di Pietro, Antonio Belli, William Palin, David J. Davies, Zubair Ahmed

**Affiliations:** ^1^ Neuroscience and Ophthalmology, Department of Inflammation and Ageing School of Infection, Inflammation and Immunology, University of Birmingham Edgbaston Birmingham UK; ^2^ NIHR Surgical Reconstruction and Microbiology Research Centre University Hospitals Birmingham Birmingham UK; ^3^ Phototherapy Research Group School of Dentistry, University of Birmingham Birmingham UK; ^4^ School of Dentistry, University of Birmingham Birmingham UK; ^5^ Centre for Trauma Sciences Research, University of Birmingham Edgbaston Birmingham UK

**Keywords:** functional recovery, medical devices, neuroprotection, photobiomodulation, traumatic brain injury

## Abstract

Mild traumatic brain injury (mTBI) is a common consequence of head injury but there are no recognized interventions to promote recovery of the brain. We previously showed that photobiomodulation (PBM) significantly reduced the number of apoptotic cells in adult rat hippocampal organotypic slice cultures. In this study, we first optimized PBM delivery parameters for use in mTBI, conducting cadaveric studies to calibrate 660 and 810 nm lasers for transcutaneous delivery of PBM to the cortical surface. We then used an in vivo weight drop mTBI model in adult rats and delivered daily optimized doses of 660, 810 nm, or combined 660/810 nm PBM. Functional recovery was assessed using novel object recognition (NOR) and beam balance tests, whilst histology and immunohistochemistry were used to assess the mTBI neuropathology. We found that PBM at 810, 660 nm, or 810/660 nm all significantly improved both NOR and beam balance performance, with 810 nm PBM having the greatest effects. Histology demonstrated no overt structural damage in the brain after mTBI, however, immunohistochemistry using brain sections showed significantly reduced activation of both CD11b^+^ microglia and glial fibrillary acidic protein (GFAP)^+^ astrocytes at 3 days post‐injury. Significantly reduced cortical localization of the apoptosis marker, cleaved caspase‐3, and modest reductions in extracellular matrix deposition after PBM treatment, limited to choroid plexus and periventricular areas were also observed. Our results demonstrate that 810 nm PBM optimally improved functional outcomes after mTBI, reduced markers associated with apoptosis and astrocyte/microglial activation, and thus may be useful as a potential regenerative therapy.

**Translational Impact Statement**

Photobiomodulation (PBM) may stimulate neuroprotection after mild traumatic brain injury (mTBI). We show the development and use of transcutaneous PBM in preclinical models of mTBI. Our results demonstrated that transcutaneous PBM improved functional recovery, reduced astrocyte and microglial activation, reduced the apoptosis marker, cleaved caspase‐3, after mTBI. PBM therefore has the potential to be neuroprotective and improve functional recovery in patients with mTBI.

## 1 INTRODUCTION

Traumatic brain injury (TBI) is a significant global health problem and a common cause of death and disability, particularly in young people.^1^ Though the consequences of moderate and severe injuries can be profound, even mild injuries (i.e., mTBI) often result in persistent disabling symptoms: over 50% of those injured report ongoing symptoms persisting for more than 6 months after injury, including headaches, depression, cognitive symptoms, and balance problems.^2^, ^3^, ^4^, ^5^ Despite the clinical need for interventions to improve recovery, no effective disease‐modifying therapy has been identified to control the variety of post‐injury responses and prevent secondary injury to brain tissue, and ensuing cell damage/loss.^6^, ^7^

Prospective TBI therapeutics must successfully intervene across a multitude of pathophysiological mechanisms to influence patient outcome.^7^ Photobiomodulation (PBM) is an optical therapy based on the delivery of red or near‐infrared wavelength light (600–1000 nm) for a therapeutic effect.^8^, ^9^ The mechanism of action of PBM is thought to be based on the chromophore activity of cytochrome c oxidase, as this complex of the electron transport chain readily absorbs such light energy.^10^, ^11^ This has numerous downstream effects within the mitochondria via stabilization of mitochondrial membrane potential,^12^ which averts the overproduction of reactive oxygen species by dysfunctional mitochondria, reducing local oxidative stress.^13^ By modulation of apoptotic pathways,^14^, ^15^, ^16^, ^17^ cell survival is increased and PBM has also been demonstrated to reduce microglia activation after TBI, and thus ameliorate the local inflammatory response.^18^ Pre‐clinical studies on the efficacy of PBM in acute TBI have generated positive results, utilizing moderate or severe models of TBI in rodent species,^9^ with promising results in an early clinical study.^19^

Early clinical applications of PBM have shown promise for beneficial effects on functional and neuropsychiatric symptoms in the rehabilitative phase of TBI.^20^ Similar pilot work in concussion/mTBI has also yielded encouraging improvements of symptomatology.^21^, ^22^, ^23^ There is also a growing body of evidence for the beneficial effects of PBM in tauopathies such as Alzheimer's disease^24^, ^25^; such beneficial effects may also translate to TBI in the prevention or therapy for chronic traumatic encephalopathy. As described previously, meta‐analysis for neurological severity score and lesion size were found to favor intervention versus control.^9^ Sub‐group analysis based on PBM parameter variables for these outcomes showed that favorable parameters were identified as wavelengths in the region of 665 and 810 nm; time to first administration of PBM ≤4 h; and total number of daily treatments ≤3. No differences were identified between pulsed and continuous wave modes or energy delivery. As such, key features of the parameters were determined as: use of 660 and/or 810 nm PBM; and first application within 4 h, continuing for up to 3 days.

We have also previously described the effect of 660 nm PBM in organotypic hippocampal slice cultures,^26^ demonstrating optimal efficacy of PBM when delivered at average irradiances of 21–42 mW/cm^2^. This serves as important in vitro precedence for use of average irradiance values within this range for application in TBI but requires further validation in vivo. The relative benefits of 660 versus 810 nm PBM are less clear. However, 810 nm offers improved penetrance through superficial tissues,^27^ though little is known on the difference, if any, in chromophore for these wavelengths,^28^ and their use in combination may provide a compound benefit in comparison to a single wavelength alone. The current study aimed to (1) validate the delivery of PBM in the region of 20–40 mW/cm^2^ to the cortical surface of the rat brain using ex vivo modeling; and (2), assess the efficacy of varying wavelengths of PBM to promote functional and histological recovery after mTBI.

## 2 MATERIALS AND METHODS

All animal procedures were licensed by the UK Home Office and approved by the University of Birmingham's Animal Welfare and Ethical Review Board (PPL number: PP6036294; protocol 2). Surgeries were carried out in strict accordance with the guidance of the UK Animals Scientific Procedures Act, 1986. Power calculations using the NC3Rs Experimental Design Assistant were set up to achieve a statistical significance α *p* <0.05 with a power of 0.8. Effect sizes and standard deviations were based on similar studies in a spinal cord injury (SCI) model,^29^ obtaining sample sizes of *n* = 4–5 rats/group. An effect size of at least 30% improvement in novel object recognition (NOR) and beam crossing was determined as clinically relevant. Variability for outcome measures was determined from unpublished pilot experiments in establishing the model and behavioral measures in our centre. All animals were randomly assigned to treatment groups and masked to the experimenters until analysis was complete. Pre‐ and post‐operative analgesia was provided as standard and as advised by the named veterinary surgeon.

### 2.1 Ex vivo cadaveric modeling and profilometry

Adult (300–350 g) male Wistar rats (Charles River, Margate, UK) were sacrificed by exposure to rising concentrations of CO_2_, and the heads removed, frozen, and stored at −20°C until use. On use, heads were allowed to thaw and after shaving of the scalp, heads were cut using a 24tpi hacksaw parallel along an anatomical plane from the lateral canthus to the external acoustic meatus. The residual brain was removed, and the sample was positioned with the scalp in direct contact with the laser probe surface, with power meter/beam profile focused at the inner skull surface. Lasers used were 660 nm MDL‐III‐660‐FC‐800 mW and 810 nm MDL‐III‐810‐FC‐800 mW, (both from CNI Co Ltd., Changchun, China), coupled to a custom fiber patch cable (FG550UEC, Thor Labs, USA).

Profilometry was performed using the methods described by us previously.^29^ Beam characterization was performed with an SP620 camera and BeamGage Standard software (6.17), (both from Ophir Photonics, Munich, Germany). Neutral density filters were used where required to optimize characterization within the tolerances of the SP620 to avoid sensor saturation (ND1/2, Ophir Photonics). Linear calibration was performed, and ambient light calibration was performed using the Ultracal function of the integrated BeamGage software. 𝐷4𝜎 values were derived from beam diameters using the integrated Autoaperture function. For direct beam profiling the SP620 camera focused on a target screen (LMR2/M, Thor Labs, Newton, NJ, USA) at a specified distance from the emitting fiber. Power calibration values were derived through two methods. For spectral irradiance, spectrometry was acquired with a USB4000 spectrometer coupled to a 200 μm fiber with a CC‐3‐UV‐T cosine corrector for an inlet aperture of 400 μm (all from OceanOptics, Ostfildern, Germany). The spectrometer was calibrated using a standard halogen light source (DH‐2000, OceanOptics). Spectral irradiance was calculated via AUC integration calculation (Microsoft Excel, Microsoft, USA). Where aperture or sensitivity range of spectrometer system was not compatible with measurement, power values were acquired with PD300R‐3 W power meter (10 mm aperture, used with manufacturer‐supplied removable filter) and StarLab 3.0 software (all from Ophir Photonics).

For thermal imaging of the temperature changes associated with light administration, an AX5 (Teledyne FLIR LLC, Oregon, USA) camera was focused onto the inner surface of the skull. Image capture and thermal profiling were performed using FLIR ResearchIR (Teledyne FLIR, Wilsonville, OR, USA). Tissue surfaces were assigned an emissivity of 0.96.^30^

### 2.2 Weight drop model of mild traumatic brain injury

Adult (300–350 g, 12–15 weeks of age) male Wistar rats (Charles River) were anesthetized using 5% isoflurane with 1.8 L/min O_2_ after administration of intraperitoneal buprenorphine. For the procedure, anesthesia was maintained using 2.5% isoflurane. Weight drop injury was performed as described previously.^31^, ^32^, ^33^ Briefly, a midline incision was made in the scalp, with lateralization of the periosteum to expose the skull. A cyanoacrylate adhesive was used to temporarily secure a 10 mm diameter 5 mm thickness protective metal (steel) disc to the surface of the skull, centered on the bregma. Animals were then transferred to a thick polyurethane foam block and positioned directly under a clear plastic guide tube containing a 450 g metal weight suspended at 1 m height. The weight was released and fell under gravity to impact the metal disc directly. Animals were then returned to the operative field for inspection of the skull to ensure no fracture or hemorrhage had occurred. After closure of the wound, a standard area of the scalp (described below) was exposed to either PBM or sham (ambient room light) therapy. Control (uninjured) animals that did not receive a weight drop injury were also used. *n* = 4–5 animals/group were used.

### 2.3 Application of photobiomodulation

After injury, animals were randomized to the treatment group and PBM was delivered daily (days 0, 1, and 2) commencing 10 min post‐injury, for 2 min per session (1 min per hemisphere) at 20 mW/cm^2^ at the level of the cortex (2.4 J). Average irradiance was normalized to the respective wavelength (described below). For each hemisphere, the laser probe was positioned for 10 s on a map of six adjacent points to avoid accumulation of heating in the skin. For 660/810 nm protocol, 1.2 J of PBM was delivered over 1 min (30 s per hemisphere) each for 660 and 810 nm (5 s per point). Subsequent applications of PBM were administered to the same marked six sites without anesthesia and with animals held in place through gentle restraint and manual positioning of the PBM probe. For sham treatment, the same process was performed with the source end of the laser probe exposed to ambient room light.

This protocol was selected based upon the findings of our previous systematic review of pre‐clinical studies and the effective parameters identified in a meta‐analysis.^9^ This identified that efficacy was most significant based on: ≤3 treatments; commencing within 4 h of injury; and using either 660 or 810 nm. A broad range of radiant exposures per dose have been effective in vivo in previous studies.^9^ Therefore, we utilized dosing studies from our previous works which identified that radiant exposures in the region of 2.4 J/cm^2^ (60–120 s using 20–40 mW/cm^2^).^26^, ^29^

### 2.4 Novel object recognition test

For assessment of spatial memory and exploratory behavior, novel object recognition (NOR) was performed as described elsewhere.^34^, ^35^, ^36^, ^37^ All habituation and testing were performed in the home cage, with other rats temporarily moved to an identical alternative cage. Five days prior to injury, rats were habituated to the task with no data recorded. With individual rats, two large identical objects were placed randomly in each half of the cage, and each rat was given 3 min to freely explore the objects. For testing post‐injury, rats were again habituated to these two, identical, familiar objects for 3 min. After 1 h, rats were then returned to the cage, where one familiar object had been replaced with a markedly different “novel” object. Over a 3 min test period, time spent interacting with the novel object was recorded by two masked observers independently and averaged over the two readings. The entire process of habituation and testing was repeated weekly for 4 weeks.

### 2.5 Beam‐walking test

For assessment of balance and motor recovery, beam‐walking (BW) was performed as described elsewhere.^34^, ^38^, ^39^, ^40^ Training was performed 4–5 days prior to administration of injury. For testing, rats were placed on a 1.2 m long 25 mm diameter cylindrical rod traversing two raised platforms. The rod was fixed within mounts and did not rotate. A cage was accessible at one end, with a lamp positioned at the other to encourage beam walking. Over three trials, interspersed with rest periods of 1 min, two masked observers independently scored the number of slips/errors and successful steps and averaged over the two observers. Testing was recorded on three occasions over 4 weeks (every 8 days).

### 2.6 Tissue processing

After completion of the behavioral tests, the same animals were sacrificed and intracardially perfused with 4% formaldehyde. Whole brains were removed and post‐fixed in 4% formaldehyde before cryoprotection in graded sucrose solutions. For embedding in OCT mounting media (Avantor, Radnor, PA, USA), 5 mm of the anterior pole of the brain was removed with a single coronal slice, for mounting on the flat coronal edge. Coronal brain sections were cut at 15𝜇m‐thick using a cryostat (OTF, Bright Instruments, Huntingdon UK), adhered onto charged slides (SuperFrost+, Fisher Scientific, Waltham, MA, USA) and stored at −20°C until use.

### 2.7 Histology and immunohistochemistry

Histological staining was performed using a commercially available kit that contained hematoxylin and eosin (HAE‐2‐IFU, ScyTek, Utah, USA) according to the manufacturer's instructions. Briefly, samples were hydrated in distilled water before addition of Hematoxylin Mayer's (Lille's modification) for 5 min. Samples were rinsed twice and incubated with bluing agent for 15 s. After rinsing in distilled water and absolute ethanol, Eosin Y was added to tissue sections for 3 min. After rinsing in distiled water, sections were finally rinsed in three changes of absolute alcohol, cleared in Histoclear II (HS‐202, National Diagnostics, Kolkata, India), and mounted using Permount toluene mounting resin (SP15, Fisher Scientific).

Immunofluorescence staining was performed using antibodies listed in Table 1, as described previously.^29^, ^41^ Briefly, tissue sections were thawed at room temperature, washed in PBS, and permeabilized in PBS containing 0.1% Triton‐X‐100. After further washes, tissue sections were blocked in PBS containing 0.5% BSA and 0.1% Triton‐X‐100 and incubated with primary antibodies diluted in PBS containing 0.5% BSA and 0.01% Tween‐20, overnight at 4°C. Sections were then washed in PBS, incubated with appropriate secondary antibodies, washed in PBS, and mounted using Vectamount containing DAPI (Vector Laboratories, Peterborough, UK). Tissue sections correlating to −1.30 mm to the bregma were used (i.e., the injury epicenter and covered by PBM) for its broad range of anatomical regions within a single slice.

**TABLE 1:** Antibody clones used with dilution, manufacturer and catalogue number.

Antigen	Dilution	Supplier	Catalogue No.
Laminin	1:400	Sigma, Massachusetts, USA	L9393
NF200	1:400	Sigma, Massachusetts, USA	N4142
Iba1	1:200	Invitrogen, Massachusetts, USA	PA5‐27436
Fibronectin	1:200	Sigma, Massachusetts, USA	F3648
GFAP	1:200	Sigma, Massachusetts, USA	G9269
CD11b/c (OX42)	1:100	Abcam, Cambridge, UK	ab1211
CD68	1:100	Abcam, Cambridge, UK	ab283654
Cleaved caspase‐3	1:200	Cell Signaling Technology, Massachusetts, USA	9661S
Goat anti‐mouse IgG (Alexa Fluor 488)	1:400	Fisher Scientific, Loughborough, UK	A‐11011
Goat anti‐rabbit IgG (Alexa Fluor 594)	1:400	Fisher Scientific, Loughborough, UK	A‐11012

### 2.8 Image acquisition and analysis

Tissue sections were imaged and analyzed by a reviewer blinded to the treatment conditions. Predetermined brain areas were identified anatomically under a fluorescent microscope with images captured with an Axioplan epi‐fluorescence microscope equipped with an Axiocam HRc camera and controlled by Axiovision Software (all from Carl Zeiss, Welwyn‐Garden City, UK). Sections without primary antibodies were used to control for background fluorescence. Images were analyzed using ImageJ (National Institutes of Health, Bethesda, MD, USA)^42^ by applying a fixed region of interest (ROI) to determine integrated density. For regions with variable morphology (e.g., choroid plexus), the anatomical ROI was manually traced, and mean gray values acquired for comparison. For cleaved caspase‐3^+^ nuclei, counts were taken of cells >100 pixel area and circularity >0.2, after image thresholding.

### 2.9 Statistical analysis

Analysis of functional outcome data was performed using GraphPad Prism (V9.2.0 GraphPad, New York, NY, USA) by repeated measures two‐way ANOVA with Geisser–Greenhouse correction and post‐hoc Dunnett's method for multiple comparisons. Immunostaining outcomes were analyzed using one‐way ANOVA with post‐hoc Dunnett's method.

## 3 RESULTS

### 3.1 Effective doses of 660 and 810 nm PBM to the brain are achievable via transcutaneous administration in an in vivo model of mTBI

**FIGURE 1:**
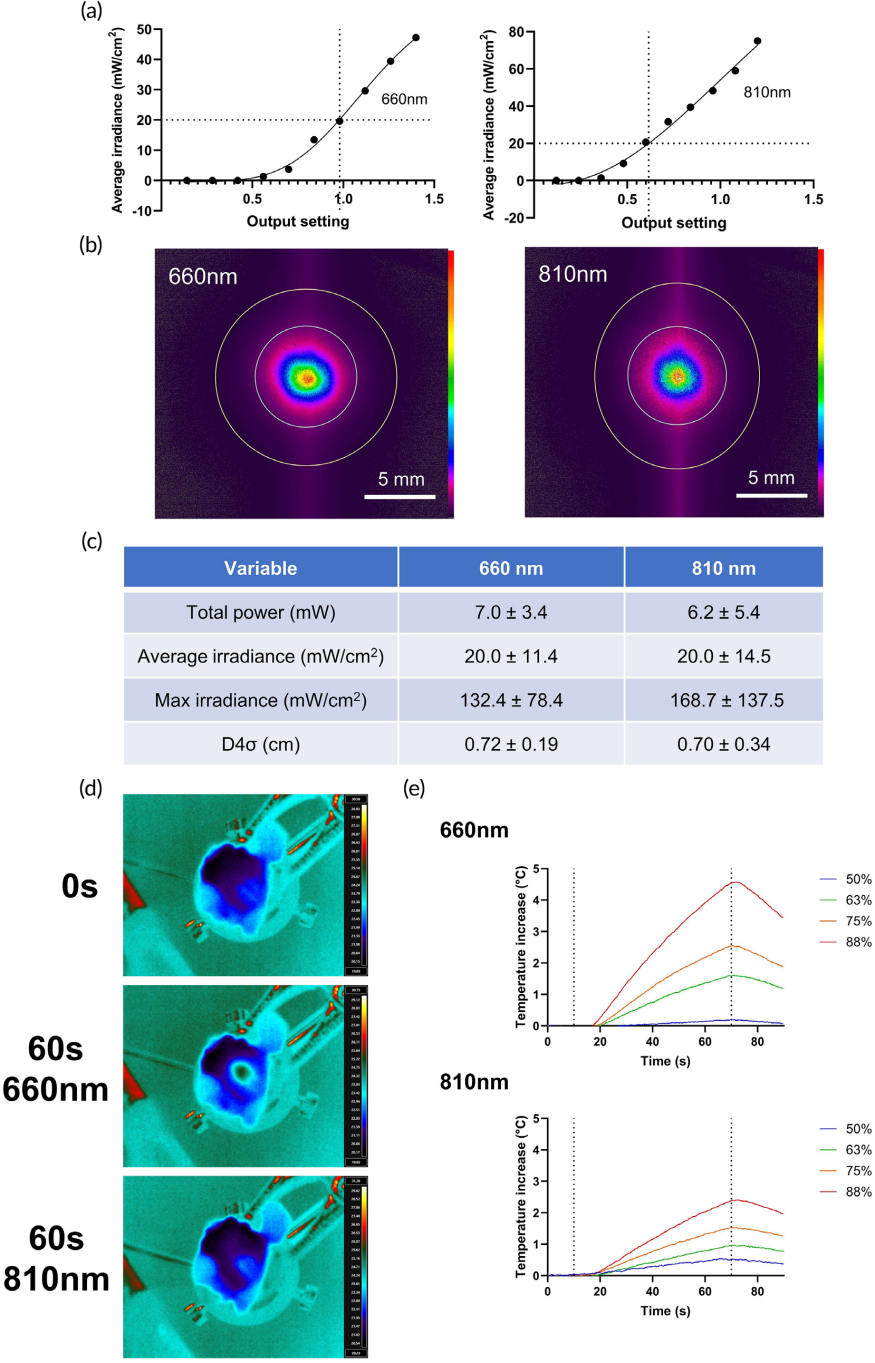
(a) Plot of average irradiance transmitted to the inner surface of skull against laser output setting for 660 (left panel) and 810 nm (right panel) lasers. Additional × axis line indicates 20 mW/cm^2^ level, with corresponding y axis line at the intersection to plot required output setting. Output settings given in manufacturer determined arbitrary units as a 0%–100% output (non‐linear) of 1.4 (660 nm) and 1.2 (810 nm). (b) Beam profiles at inner surface of skull with transcutaneous application of PBM for 660 (left panel) and 810 nm (right panel) lasers. Gaussian distribution of transmitted light with a D4𝜎 of 0.70–0.72 cm. Relative intensity color bar to right (OSI Rainbow). (c) Table showing beam profilometry readings with SD using output settings derived from (a). *n* = 4 per group. (d) Images of FLIR thermal profiling taken from the internal skull surface at 0, and 60 s during 60 s treatment protocol for 20 mW/cm^2^ 660 nm and 810 nm PBM. (e) Temporal plots of thermal change (maximum temperature) for a region of interest on the internal skull surface during 60 s treatment protocol for 660 nm and 810 nm PBM. *x*‐axis lines denote ON (10 s) and OFF (70 s), with 10 s observation to establish temperature baseline prior to PBM, and 20 s post‐administration to observe cooldown. Temperature change given is relative to background temperature recorded within the field of view of the thermal imaging camera, from tissue not exposed to PBM. *n* = 4 per group. Treatment protocols used for PBM were 70% output for 660 nm and 63% for 810 nm.

Cadaveric profiling of transcranial transmission from 660 and 810 nm probes was performed using ex vivo specimens of male Wistar rat scalps and skulls (Figure 1a–c). Profilometry demonstrated that PBM using 810 nm probes demonstrated improved transmission at lower power outputs compared with 660 nm. Calibration of transmission curves for both sources allowed matching of average irradiance to 20 mW/cm^2^ for both (70% output for 660 nm; 63% for 810 nm), whilst D4𝜎 was equivalent for each source. To ascertain safe output parameters for avoidance of significant heating, thermography of the internal skull surface during a 60s treatment showed that maximal heating was less than 2°C for both treatment output levels, with no focal heating over the first 10s (Figure 1d, e). Ex vivo temperature profiling likely overestimates the heating in vivo, due to the presence of blood flow and greater tissue volume to act as a heat sink. Nevertheless, these output levels were deemed the highest usable levels to avoid focal skin heating and are well within safe limits to avoid discomfort during short exposures. Movement of the probe across the skin every 10 s was implemented to avoid greater focal skin heating than observed at skull level. During in vivo trials, no animal discomfort was observed during a 2 min treatment protocol for either wavelength. Full profilometry at scalp and cortical levels are given in Supplementary Table 1.

### 3.2 Photobiomodulation improves functional recovery after mTBI

**FIGURE 2:**
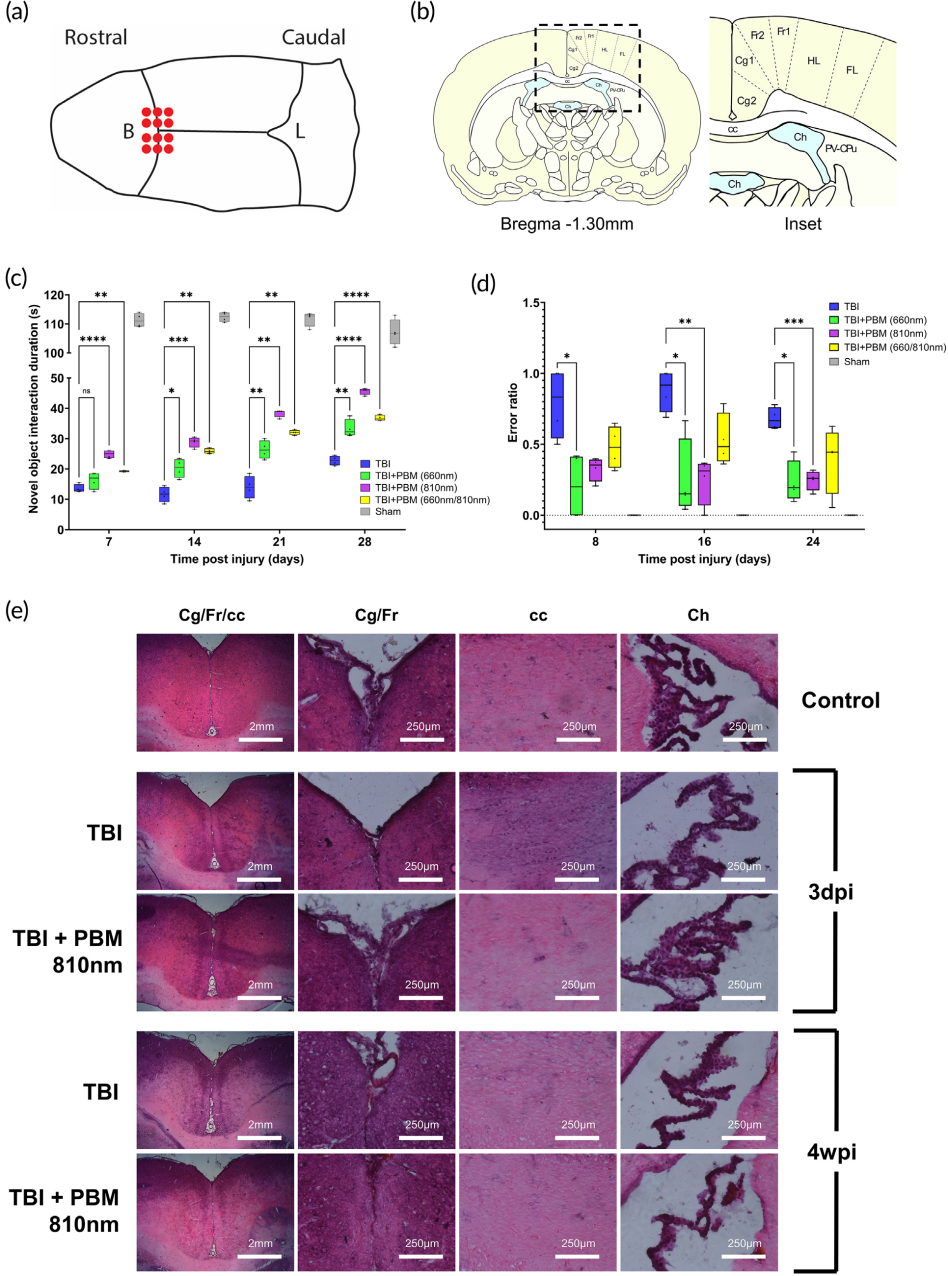
(a) Schematic diagram of a rat skull (surface view) with red dots demonstrating the 12 points of PBM administration. B = bregma; L = lambda. (b) Schematic diagram of a coronal section of adult rat brain (bregma 1.30 mm) with inset demonstrating brain areas (labeled) imaged for assessment of immunofluorescence. cc, corpus callosum; Cg1, cingulate cortex area 1; Cg2, cingulate cortex area 2; Ch, choroid plexus (within lateral and third ventricles); FL, forelimb area of cortex; Fr1, frontal cortex area 1; Fr2, frontal cortex area 2; HL, hindlimb area of cortex; PV‐CPu, periventricular region of caudate putamen. (c) Novel object recognition at four timepoints. (d) Beam walk (error ratio calculated by the formula errors/completed steps) at three time points. * = *p* <0.05 ** = *p* <0.01 *** = *p* <0.001 **** = *p* <0.0001. Comparisons for TBI vs. control not shown (all *p* <0.0001). All *n* = 4 per group. PBM, photobiomodulation; TBI, traumatic brain injury. (e) H&E staining on brain coronal slices from all treatment conditions at 3 dpi (days post‐injury) and 4 wpi (weeks post‐injury). Images were taken from frontal cortex, corpus callosum, and choroid plexus (body of the lateral ventricle). No differences evident, with the exception of some increased cellularity (hematoxylin staining) within the corpus callosum in acute (3 dpi) phase, not evident with PBM therapy at this time point. PBM taken from 810 nm groups. All *n* = 4 per group.

Application of transcutaneous PBM across 12 points over the bregma (Figure 2a, b), demonstrated significant improvements in functional recovery across all time points studies (Figure 2c, d). For example, by 4 weeks post‐injury, 810 nm treated animals improved NOR performance by 100.5% (*p* <0.0001), 660 nm improved by 46.2% (*p* <0.01), and 660/810 nm by 61.5% (*p* <0.001). Beam walk performance was also improved by 65.8% (810 nm, *p* <0.001), 63.8% (660 nm, *p* <0.05), and 42.3% (660/810 nm, *p* = 0.08). Although both 660 nm and 660/810 nm reached statistical significance in NOR, no compound benefits from utilization of both wavelengths versus 810 nm alone was determined. In beam walking tests, 660 and 810 nm performed equally well and reached statistical significance at 24 days post‐injury (dpi). However, across animals, 810 nm produced improvements with greater consistency in beam walking tests, and combined with superior performance in NOR this suggests that 810 nm alone might be the optimal therapeutic strategy, which we selected for further experiments described below.

### 3.3 Weight drop mTBI and PBM do not result in structural brain injury

Histology on coronal brain sections from all treatment conditions at 3 dpi and 4 weeks post‐injury (wpi) validated the injury paradigm by ensuring that no structural injury is evident, and confirming that PBM therapy does not result in brain structural injury (Figure 2e). No discernible evidence of structural damage was identified in any of the groups. The only difference noticed is that of mildly increased cellularity present in the corpus callosum in the 3 dpi TBI group, but not identified in the group that received 810 nm PBM for 3 dpi or in control (uninjured) samples.

### 3.4 PBM reduces astrocyte and microglial activation in the acute phase after mTBI

**FIGURE 3:**
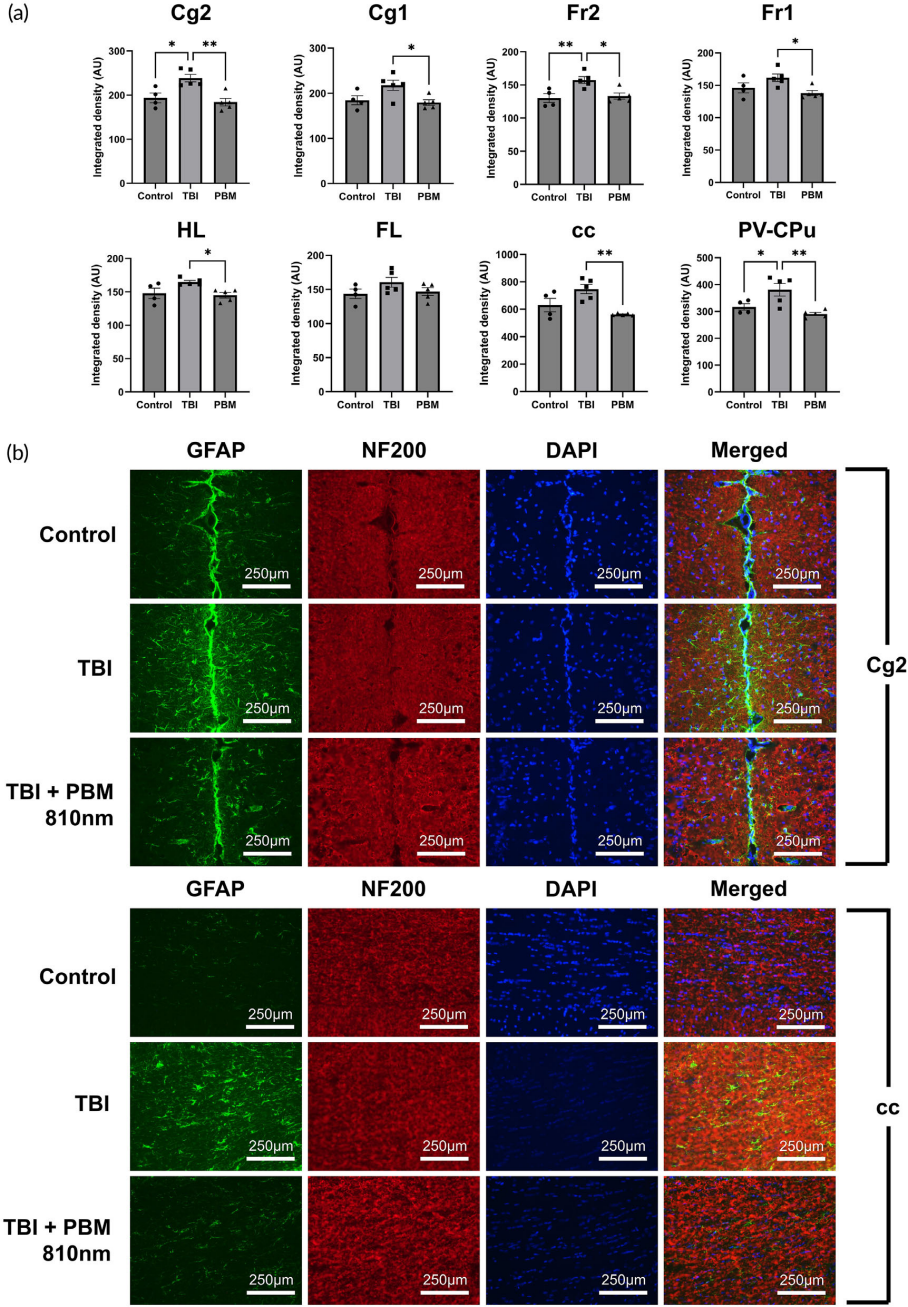
PBM reduces GFAP immunoreactivity across brain areas at 3 dpi. (A) Area controlled integrated densities for GFAP in denoted brain areas. (b) Immunofluorescence imaging for Cg2 and cc areas. A total of 810 nm used in PBM group (2 min per day (1 min per hemisphere), 20 mW/cm^2^, 2.4 J). *n* = 5 per group (*n* = 4 for control). * = *p* <0.05; ** = *p* <0.01. Non‐significant comparisons not shown. AU, arbitrary units; cc, corpus callosum; Cg1, cingulate cortex area 1; Cg2, cingulate cortex area 2; Ch, choroid plexus (within lateral and third ventricles); DAPI, 4,6‐diamidino‐2phenylindole; FL, forelimb area of cortex; Fr1, frontal cortex area 1; Fr2, frontal cortex area 2; GFAP, glial fibrillary acidic protein; HL, hindlimb area of cortex; NF200, neurofilament 200; PV‐CPu, periventricular region of caudate putamen; PBM, photobiomodulation, TBI, traumatic brain injury.

**FIGURE 4:**
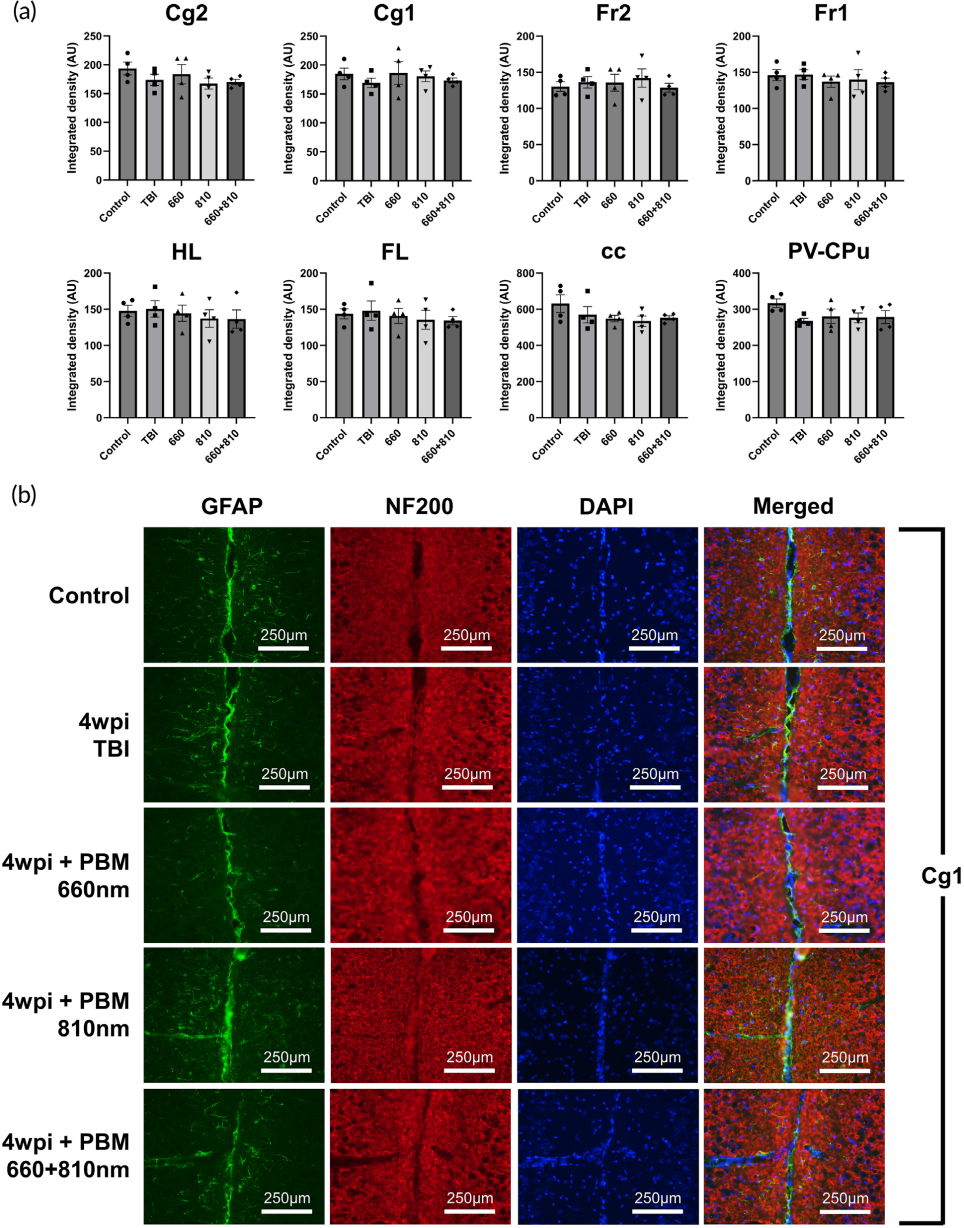
There was no discernible difference between injury, control and PBM in GFAP^+^ immunoreactive areas across brain areas at 6 wpi. (a) Area controlled integrated densities for GFAP in denoted brain areas. (b) Immunofluorescence imaging for Cg1 areas (3 dpi TBI shown for reference). A total of 660 and/or 810 nm used in PBM group (2 min per day (1 min per hemisphere), 3‐day course, 20 mW/cm^2^, 2.4 J). *n* = 5 per group (*n* = 4 for control). Non‐significant comparisons not shown. AU, arbitrary units; cc, corpus callosum; Cg1, cingulate cortex area 1; Cg2, cingulate cortex area 2; Ch, choroid plexus (within lateral and third ventricles); DAPI, 4,6‐diamidino‐2‐phenylindole; FL, forelimb area of cortex; Fr1, frontal cortex area 1; Fr2, frontal cortex area 2; GFAP, glial fibrillary acidic protein; HL, hindlimb area of cortex; NF200, neurofilament 200; PBM, photobiomodulation; PV‐CPu, periventricular region of caudate putamen; TBI, traumatic brain injury.

At 3 dpi, 810 nm PBM treatment correlated with significant reductions in immunofluorescence intensity across seven of the eight imaged brain areas (all except FL) (Figure 3a; *p* <0.05 to *p* <0.01). The most significant reductions in GFAP^+^ immunoreactivity were observed in the cingulate cortex area 2 and the corpus callosum (Figure 3b). However, no differences in GFAP immunoreactivity were observed between any groups at any of the regions at 4 wpi (Figure 4a, b).

**FIGURE 5:**
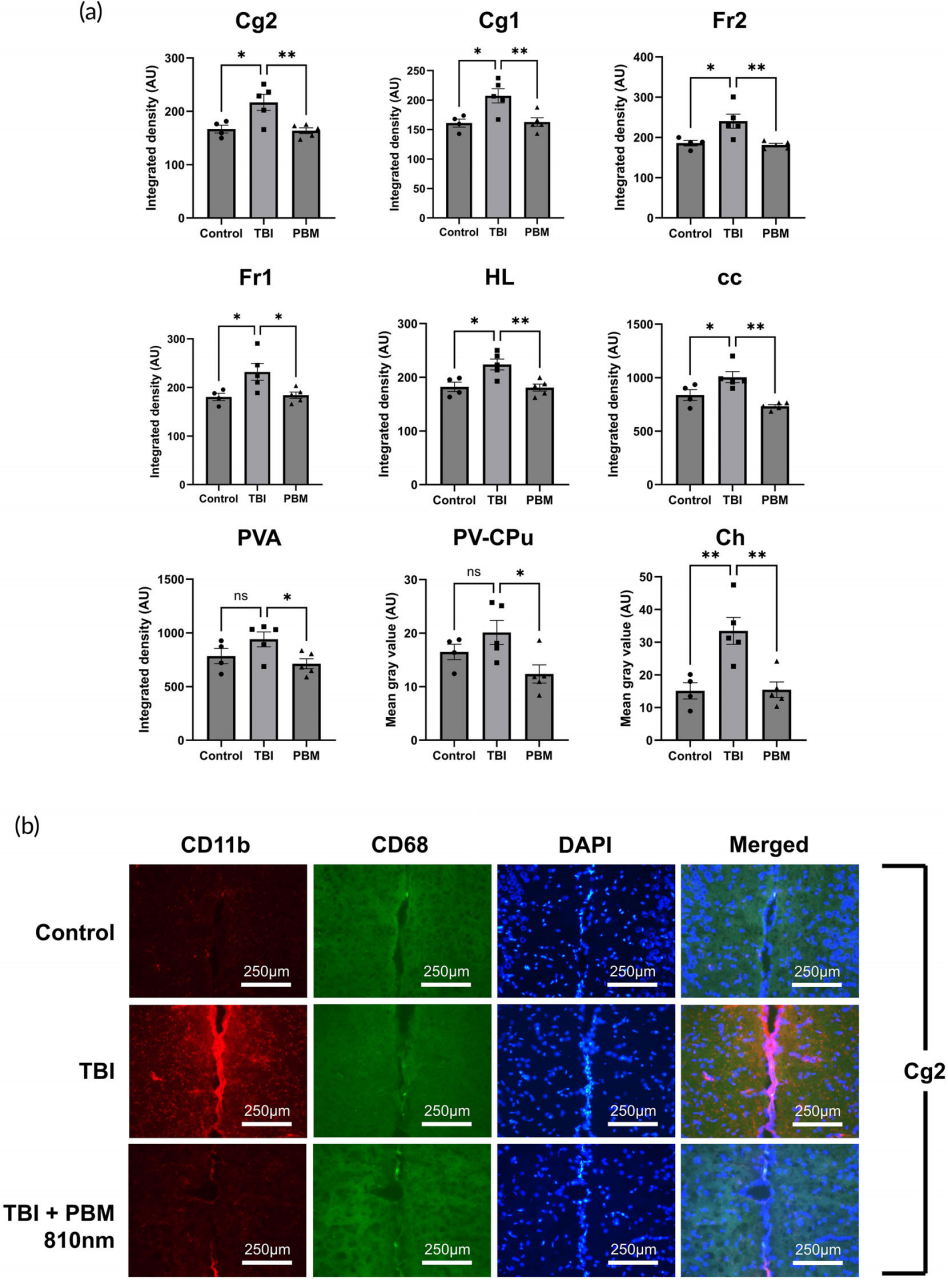
PBM reduces CD11b immunofluorescence across brain areas at 3 dpi. (a) Area controlled integrated densities for CD11b in denoted brain areas. (b) Immunofluorescence imaging for Cg2 areas. A total of 810 nm used in PBM group (2 min per day (1 min per hemisphere), 20 mW/cm^2^, 2.4 J). *n* = 5 per group (*n* = 4 for control). * = *p* <0.05; ** = *p* <0.01; ns = not significant. Non‐significant comparisons not shown. cc, corpus callosum; AU, arbitrary units; Cg1, cingulate cortex area 1; Cg2, cingulate cortex area 2; Ch, choroid plexus (within lateral and third ventricles); DAPI, 4,6‐diamidino‐2‐phenylindole; Fr1, frontal cortex area 1; Fr2, frontal cortex area 2; GFAP, glial fibrillary acidic protein; HL, hindlimb area of cortex; NF200, neurofilament 200; PBM, photobiomodulation; PVA, paraventricular thalamic nucleus, area a; PV‐CPu, periventricular region of caudate putamen; TBI, traumatic brain injury.

CD11b staining was elevated across all cortical areas, corpus callosum, and choroid plexus between control and TBI specimens. In the same areas, application of PBM was associated with a significant reduction in CD11b immunoreactivity (Figure 5a,b). Co‐staining for CD68 however, demonstrated no increase in CD68^+^ immunoreactivity across the different regions, with validation of technical success by CD68^+^ cells with monocytic morphology within blood vessels (Figure 5b). These results suggest that PBM reduces astrocyte and microglial activation in the injured brain in the acute phase post‐injury.

### 3.5 PBM modestly reduces extracellular matrix deposition after mTBI

**FIGURE 6:**
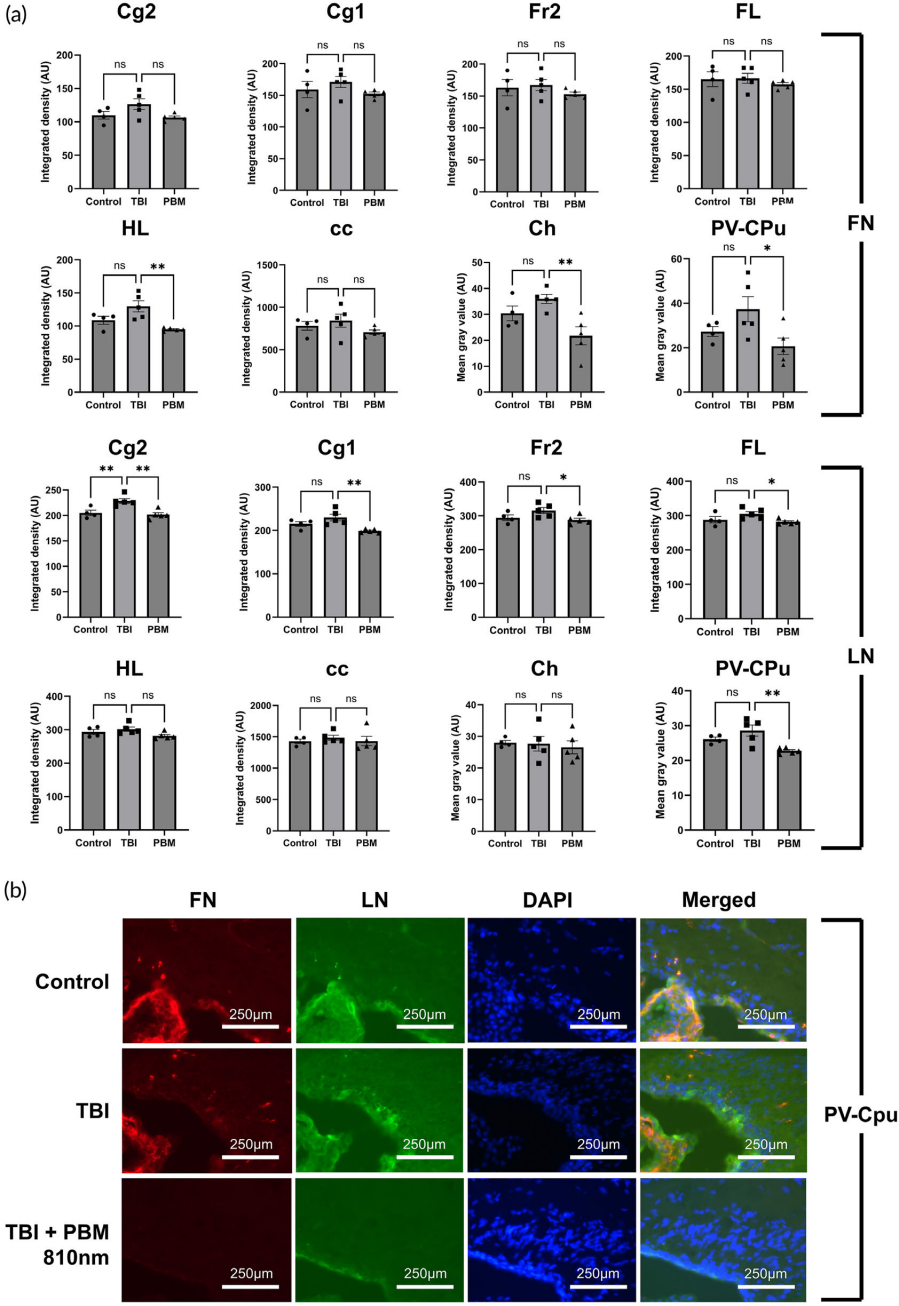
PBM results in minimal reductions in fibronectin immunofluorescence across brain areas at 3 dpi. (a) Area controlled integrated densities for fibronectin (FN) and laminin (LN) in denoted brain areas. (b) Immunofluorescence imaging for PV‐CPu areas. A total of 810 nm used in PBM group (2 min per day (1 min per hemisphere), 20 mW/cm^2^, 2.4 J). *n* = 5 per group (*n* = 4 for control). * = *p* <0.05; ** = *p* <0.01; ns = not significant. AU, arbitrary units; cc, corpus callosum; Cg1, cingulate cortex area 1; Cg2, cingulate cortex area 2; Ch, choroid plexus (within lateral and third ventricles); DAPI, 4,6‐diamidino‐2phenylindole; FL, forelimb area of cortex; FN, fibronectin; Fr2, frontal cortex area 2; GFAP, glial fibrillary acidic protein; HL, hindlimb area of cortex; LN, laminin; NF200, neurofilament 200; PBM, photobiomodulation; PVA, paraventricular thalamic nucleus, area a; PV‐CPu, periventricular region of caudate putamen; TBI, traumatic brain injury.

Brain sections were immunostained for the presence and distribution of extracellular matrix (ECM) proteins laminin and fibronectin. No significant differences between control and TBI animals were identified in any brain area, with the exception of modest differences in laminin expression in Cg2 (Figure 6a, b). Variable but significant reductions in ECM deposition were identified in PBM‐treated animals at 3 dpi (fibronectin: Ch (*p* <0.01), PV‐CPu (*p* <0.05), and HL (*p* <0.01); laminin: Cg2 (*p* <0.01), Cg1 (*p* <0.01), Fr2 (*p* <0.05), FL (*p* <0.05), and PV‐CPu (*p* <0.01)) but at 4 wpi, these observed effects were not present (Figure S1).

### 3.6 PBM reduces cleaved caspase‐3^+^ staining in the acute phase after mTBI

**FIGURE 7:**
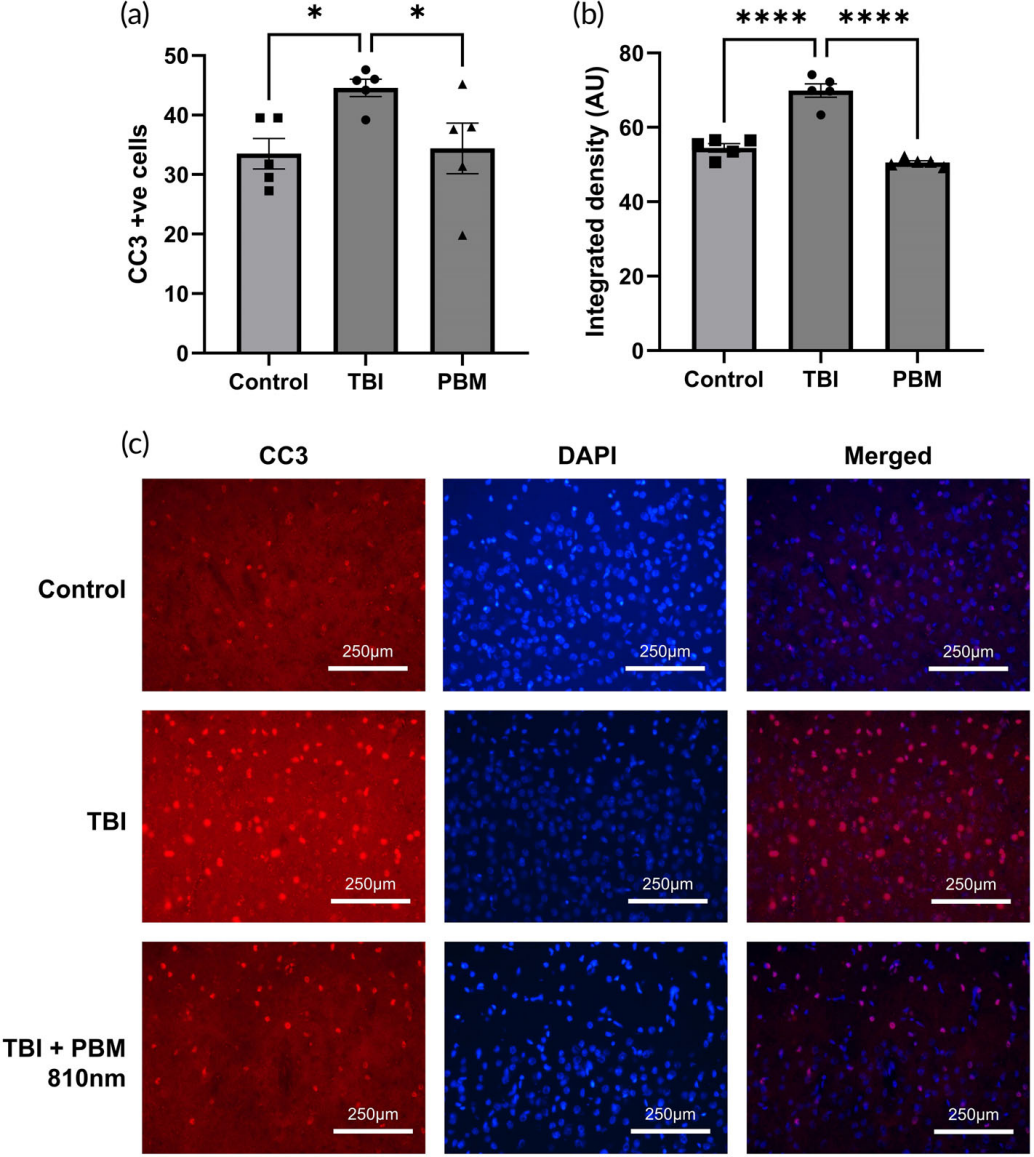
PBM results in reduced cortical expression of cleaved caspase‐3 at 3 days post‐injury. (a) Comparison of counts of cleaved caspase‐3^+^ nuclei across five cortical areas. (b) Comparison of fluorescence intensity of cleaved caspase‐3 across whole field in five cortical areas. (c) Immunofluorescence imaging in HL area. A total of 660 and/or 810 nm used in PBM group (2 min per day (1 min per hemisphere), 20 mW/cm^2^, 2.4 J/cm^2^). *n* = 4 per group. * = *p* <0.05. AU, arbitrary units; CC3, cleaved caspase‐3; DAPI, 4,6‐diamidino‐2‐phenylindole; ns, not significant; PBM, photobiomodulation; TBI, traumatic brain injury.

Cleaved caspase‐3^+^ immunostaining was used to assess levels of apoptotic activity in the cortex (areas Cg1, Cg2, Fr1, Fr2, and FL). The significantly increased number of cleaved caspase‐3^+^ cells after TBI (*p* <0.05, vs. controls), was significantly reduced by PBM treatment (*p* < 0.05, vs. TBI) (Figure 7a). Based on integrated density of fluorescence, there was a strongly significant (*p* <0.0001) increase in cleaved caspase‐3 expression after TBI compared to controls and this was again significantly reduced (*p* <0.0001) in PBM‐treated animals (Figure 7b, c). These results demonstrated that PBM reduced cleaved caspase‐3 activation, a marker of apoptosis.

## 4 DISCUSSION

This work has demonstrated the efficacy of transcranial delivery of 810 nm 20 mW/cm^2^ PBM for the improvement of functional and histological outcomes after mTBI. Adminsitration of 810 nm PBM demonstrated the greatest efficacy in improving functional outcomes and was superior to irradiance‐matched administration of either 660 nm or 660/810 nm combined PBM. Histological analysis at 3 dpi demonstrated that 810 nm PBM reduced astrocyte and microglial activation, and ECM deposition in the acute phase after mTBI and was associated with a reduction in expression of the pro‐apoptotic marker, cleaved caspase‐3, supporting a neuroprotective effect of PBM in TBI.

### 4.1 Wavelength selection

Using an in vivo model, comparison of 660 and 810 nm wavelengths, either alone or in combination, demonstrated superiority of 810 nm in NOR recovery, with equivocal efficacy of 660 and 810 nm in BW recovery. Combination of 660 and 810 nm PBM has been shown to offer differing and advantageous effects in improving brain metabolism compared to use of 810 nm alone, though such an effect was not observed here.^43^ The greater efficacy of 810 nm in vivo may be attributable to the increased penetrance of 810 nm.^27^ Although 660 and 810 nm PBM treatments were matched at 20 mW/cm^2^ at the cortical surface, the increased penetrance of 810 nm PBM likely resulted in greater irradiances reaching subcortical areas and may have contributed to the superior effects observed in vivo.

The theoretical advantage of a combination therapy is that, if complementary wavelengths target different chromophores, then favorable activity may be stimulated by their use together, eliciting a compound effect over that of activation of a single chromophore via one wavelength. However, it is known that 660 and 810 nm both activate a principal PBM target chromophore, cytochrome c oxidase (CCO).^44^ As such, this finding tentatively supports the primary role of CCO as a key mediator in the mechanism of the action PBM after TBI. Nevertheless, synergistic effects of combined wavelengths have been observed in other pathologies, and the concept warrants further exploration in neurotrauma.^45^

### 4.2 Functional outcome

The literature describes considerable variability in the recovery period after mTBI in rodent models, with outcomes at 28 dpi ranging from complete functional recovery to significant persisting deficits.^46^, ^47^ Here, at 28 dpi, no group had returned to uninjured levels of function for cognitive or vestibulomotor outcomes, though all groups demonstrated trends toward recovery over the experimental period. This recovery was accelerated by exposure to PBM compared with sham treatment.

Functional deficits that persist beyond other biochemical measures of recovery are well recognized in mild weight‐drop injury models.^48^, ^49^, ^50^, ^51^, ^52^, ^53^, ^54^ After mild weight drop injury in Sprague‐Dawley rats, persistent deficits were identified in open field testing after a single mild injury compared to uninjured controls.^48^ Single mild TBI has also been shown to result in persistent deficits at 8 and 18 weeks in elevated plus maze, Morris water maze, and Rotorod balance function.^49^ Similarly, ongoing functional deficits at or beyond 30 days post‐injury have been identified in a variety of outcome assessments, including NOR and BW, in mice models using mild weight drop‐induced TBI.^50^, ^51^, ^52^, ^53^, ^54^

### 4.3 Mechanism

Quantitative immunofluorescence imaging described here demonstrated that 810 nm PBM reduced astrocyte (GFAP) and microglial (CD11b) activation markers in the early phase post‐injury after mTBI. This effect of PBM on microglia and astrocytes has been observed in SCI.^55^, ^56^ Wang and colleagues demonstrated that this effect was mediated by PBM interfering in pro‐inflammatory crosstalk between lipocalin‐2 (Lcn2) and Janus kinases JAK/STAT3 pathways to mitigate glial activation after injury.^55^ Observations on the effects of PBM on microglial phenotype have been made previously in a variety of different models.^57^, ^58^, ^59^ The change in microglial phenotype in response to PBM is also highly dependent on fluence, with lower energies associated with anti‐inflammatory M2 phenotype and higher fluences promoting M1 pro‐inflammatory phenotypic switching.^58^ In analgesic contexts, higher energy PBM has been deployed: at these levels (8 J/cm^2^ per day) PBM is associated with an increase in microglial activation.^60^ Attenuation of monocyte activation has also been linked to reduction in A1 astrocytic activation, using conditioned media from PBM‐treated M1 macrophages, leading to reduced expression of GFAP in astrocytes.^57^

At 3 dpi, we also show that PBM is associated with reduced expression of cleaved caspase‐3. Cleaved caspase‐3 is a recognized apoptotic mediator after CNS injury,^61^, ^62^ and its gross downregulation suggests a reduction in apoptotic activity amongst all cell types in the cortex after PBM treatment. Mitochondrial damage associated with TBI results in cytochrome c release leading to caspase‐3 induction^63^ and therefore a reduction of cleaved casase‐3 activation after PBM may avert apoptosis by mitigating mitochondrial damage mediated pro‐apoptotic pathways.

Minimal changes in ECM proteins, fibronectin, and laminin, were observed between uninjured and mTBI brains. The main sites for observed changes were in the choroid plexus and periventricular caudate putamen. As the choroid plexus is a site of capillary permeability, as well as an area sensitive to damage after physical force,^64^ this is a significant area for entry of immune cells post‐injury, which may then subsequently migrate into the periventricular zone.^65^ Dampening the effects of inflammation in this region after PBM may account for the reduction of fibronectin/laminin^+^ immunoreactivity in these areas acutely after mTBI. The effects of mTBI and therapeutic interventions specific to these areas, as well as circumventricular organs, should be the subject of further investigation.

The failure of the NEST‐3 trial to show significant benefit to patients recieving PBM after ischemic stroke has highlighted the necessity for rigorous pre‐clinical optimization of PBM therapeutic protocols prior to clinical trials.^66^ Given the breadth of possible parameters for PBM treatment, more extensive optimization is required than in comparable studies using pharmaceutical treatments. Delivery modalities should be considered alongside wavelength and dosimetry variables as a key feature of PBM which can considerably affect its efficacy in treating the injured CNS.

### 4.4 Limitations

Whilst there are advantages of the use of an mTBI model in demonstrating the therapeutic benefits of PBM, the pathophysiology of moderate to severe injury significantly differs from that of mTBI and limits the scope for extrapolating these findings to more severe injury types. Similarly, as a diffuse injury model, use of a mild weight drop injury does not help to establish the effects of PBM on other facets of TBI observed clinically, such as contusional lesions.^9^ Nonetheless, since the majority of cases in humans are mTBI, the effects of PBM observed by us in this study should translate to the category of TBI with the greatest numbers of patients affected.

Due to the validation of the model previously only in male specimens, these experiments were conducted only in male subjects. Males were primarily chosen since TBI mostly affects males but it is important to recognize the possibility of sex differences in the response to PBM, and this should be a focus of future work. The immunofluorescence staining in this work has focused primarily on inflammatory, apoptotic, and astrogliotic mechanisms. However, it is important to note that PBM has been associated with synaptogenesis and neurogenesis in other published studies.^9^ Similarly, PBM is associated with significant favorable metabolic effects in acute injury states, and further exploration of such mechanisms between 660 and 810 nm PBM may offer insights into other differences in potential efficacy of these wavelengths in TBI.

Here we have calibrated delivery of 660 and 810 nm PBM for matched irradiance at the cortical surface. However, it should be noted that it is well recognized that there are significant biological effects from photobiological interactions between passing PBM photons and all tissue types encountered *en passant* through transcranial application, including scalp, bone, blood vessels, and dura. Furthermore, the effects of administration of PBM to remote target types (such as the femur or abdomen for TBI) have been shown to demonstrate favorable neuroprotective properties, particularly in the field of preventing neurodegeneration in Parkinson's disease.^67^, ^68^, ^69^ Whilst these administration modalities have been shown to be effective, the focus in this work has been on the direct effect on injured tissues. However, owing to the transcranial application of PBM, there will be PBM absorption and ensuing biological effects within superficial tissues which should not be overlooked.

Although 660 and 810 nm average irradiance values were matched using cadaveric modeling, this is not fully representative of the transmission of light in vivo, principally due to the differing absorptions of these wavelengths by both hemoglobin and water, which would differ in the in vivo state. Whilst blood was intentionally not drained post‐mortem, the absorption and scatter caused by blood in vivo would differ from the effects in any residual, deoxygenated, and coagulated blood retained in this post‐mortem model. Similarly, penetrance of PBM to depths below the brain surface is dependent on wavelength and cannot be controlled. As the aim of this modeling was to calibrate scalp outputs to achieve matched cortical dosing for 660 and 810 nm, this has not assessed the subcortical distributions owing to differing scatter by brain tissue. Cadaveric modeling represents a further limitation, in that the results of thermography during administration ex vivo will likely be a significant over‐representation of any heating occurring in vivo. Owing to continual blood flow, increased baseline temperatures, and larger volumes of tissue all acting as a “heat‐sink” in the in vivo setting, heating would be expected to be much less during administration to living tissue as these factors were not represented during ex vivo modeling.

## 5 CONCLUSION

In conclusion, this work provides significant evidence on the efficacy of PBM in mTBI, and importantly the advantage of 810 nm as the most effective wavelength for promoting recovery. This work has also demonstrated insights into the mechanisms by which PBM can mitigate damage and promote repair after neurotrauma. PBM appears to be a viable therapy after neurotrauma, that not only promotes neuroprotection but also contributes to recovery of lost function.

## AUTHOR CONTRIBUTIONS

**Andrew R. Stevens:** Writing – original draft; writing – review and editing; funding acquisition; visualization; project administration; data curation; formal analysis; validation; methodology; conceptualization; investigation. **Mohammed Hadis:** Software; supervision; resources; methodology; conceptualization; validation; funding acquisition; writing – review and editing. **Abhinav Thareja:** Investigation; validation; writing – review and editing. **Freya G. Anderson:** Writing – review and editing; investigation; validation. **Michael R. Milward:** Conceptualization; funding acquisition; methodology; writing – review and editing; supervision; resources. **Valentina Di Pietro:** Investigation; validation; methodology; writing – review and editing; resources. **Antonio Belli:** Resources; supervision; project administration; writing – review and editing; funding acquisition; conceptualization; methodology. **William Palin:** Conceptualization; methodology; funding acquisition; writing – review and editing; resources; supervision; project administration. **David J. Davies:** Supervision; conceptualization; methodology; writing – review and editing; funding acquisition. **Zubair Ahmed:** Resources; supervision; data curation; formal analysis; project administration; visualization; validation; methodology; writing – review and editing; funding acquisition; investigation; conceptualization; software.

## FUNDING INFORMATION

This study was funded by the Medical Research Council, grant number MR/X502996/1.

## CONFLICT OF INTEREST STATEMENT

Members of the authorship have submitted a patent pending application (DD, MH, WP, AS, and ZA) relating to the invasive delivery of PBM (UK Patent App. No. 2006201.4; US Patent App. 17/922, 157, 2023). There are no other competing interests to declare, including those relating to employment, consultancy, other patents, or products in development.

### PEER REVIEW

The peer review history for this article is available at https://www.webofscience.com/api/gateway/wos/peer-review/10.1002/btm2.10727.

## Supporting information


**Figure S1.** No discernible differences in fibronectin or laminin immunofluorescence were present across brain areas at 4wpi. Top panel: area controlled integrated densities for fibronectin (FN) and laminin (LN) in denoted brain areas. Bottom panel: immunofluorescence imaging for PV‐CPu areas. 660 and/or 810 nm used in PBM group (2 min per day (1 min per hemisphere), 20 mW/cm^2^, 2.4 J). *n* = 4 per group. * = *p* < 0.05. Non‐significant comparisons not shown. cc = corpus callosum; Cg1 = cingulate cortex area 1; Cg2 = cingulate cortex area 2; Ch = choroid plexus (within lateral and third ventricles); FL = forelimb area of cortex; Fr2 = frontal cortex area 2; HL = hindlimb area of cortex; PVA = paraventricular thalamic nucleus, area a; PV‐CPu = periventricular region of caudate putamen; GFAP = glial fibrillary acidic protein; NF200 = neurofilament 200; DAPI = 4,6‐diamidino‐2‐phenylindole; TBI = traumatic brain injury; PBM = photobiomodulation; FN = fibronectin; LN = laminin, AU = arbitrary units.

**Supplementary Table 1.** Profilometry of 660 and 810 nm laser sources at scalp and cortical surfaces. Lasers used were 660 nm MDL‐III‐660‐FC‐800 mW and 810 nm MDL‐III‐810‐FC‐800 mW, (both from CNI Co Ltd., Changchun, China) coupled to a custom fiber patch cable (FG550UEC, Thor Labs, USA). D4σ given as average of *x* and *y* diameters.

## Data Availability

The data that support the findings of this study are available from the corresponding author upon reasonable request.
