# Supplementary figures and images for: Photobiomodulation improves functional recovery after mild traumatic brain injury
Source: Bioeng Transl Med. 2024 Oct 11;10(2):e10727. doi: 10.1002/btm2.10727 (PMC11883100; doi:10.1002/btm2.10727)

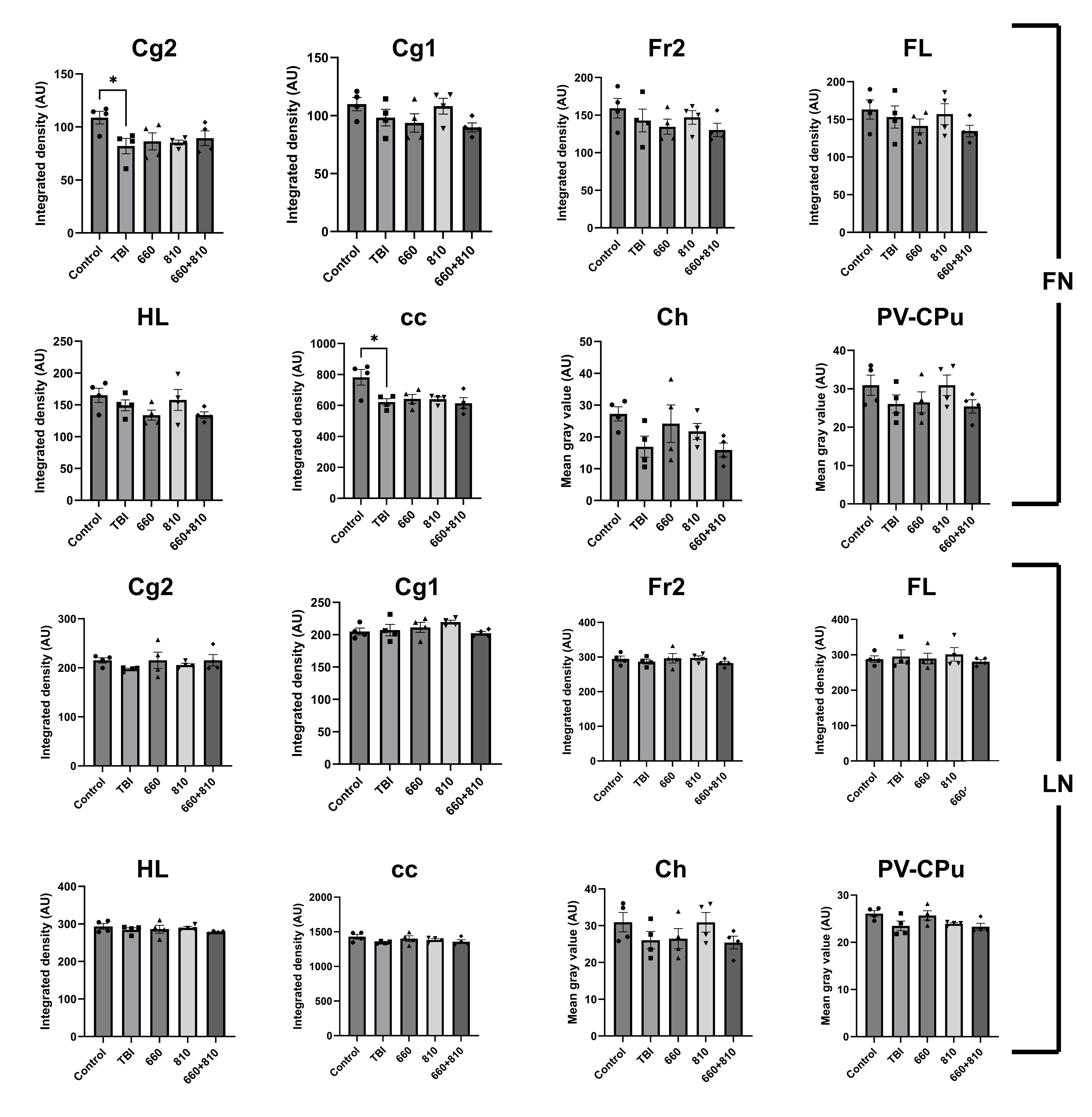

Supplement: Supplementary file 1 — Figure S1. No discernible differences in fibronectin or laminin immunofluorescence were present across brain areas at 4wpi. Top panel: area controlled integrated densities for fibronectin (FN) and laminin (LN) in denoted brain areas. Bottom panel: immunofluorescence imaging for PV‐CPu areas. 660 and/or 810 nm used in PBM group (2 min per day (1 min per hemisphere), 20 mW/cm2, 2.4 J). n = 4 per group. * = p < 0.05. Non‐significant comparisons not shown. cc = corpus callosum; Cg1 = cingulate cortex area 1; Cg2 = cingulate cortex area 2; Ch = choroid plexus (within lateral and third ventricles); FL = forelimb area of cortex; Fr2 = frontal cortex area 2; HL = hindlimb area of cortex; PVA = paraventricular thalamic nucleus, area a; PV‐CPu = periventricular region of caudate putamen; GFAP = glial fibrillary acidic protein; NF200 = neurofilament 200; DAPI = 4,6‐diamidino‐2‐phenylindole; TBI = traumatic brain injury; PBM = photobiomodulation; FN = fibronectin; LN = laminin, AU = arbitrary units. [file BTM2-10-e10727-s001.jpg]
