# Supplementary material for: Photobiomodulation improves functional recovery after mild traumatic brain injury
Source: Bioeng Transl Med. 2024 Oct 11;10(2):e10727. doi: 10.1002/btm2.10727 (PMC11883100; doi:10.1002/btm2.10727)
Supplement: Supplementary file 2 — Supplementary Table 1. Profilometry of 660 and 810 nm laser sources at scalp and cortical surfaces. Lasers used were 660 nm MDL‐III‐660‐FC‐800 mW and 810 nm MDL‐III‐810‐FC‐800 mW, (both from CNI Co Ltd., Changchun, China) coupled to a custom fiber patch cable (FG550UEC, Thor Labs, USA). D4σ given as average of x and y diameters. [file BTM2-10-e10727-s002.docx]

|  | 660 nm | | 810 nm | |
| --- | --- | --- | --- | --- |
|  | Scalp surface | Cortex | Scalp surface | Cortex |
| Total power (mW) | 51.0 ± 0.6 | 7.0 ± 3.4 | 30.5 ± 0.1 | 6.2 ± 5.4 |
| Average irradiance (mW/cm^2^) | 45,581.1 ± 463.6 | 20.0 ± 11.4 | 16,847.1 ± 61.1 | 20.0 ± 14.5 |
| Spot size (D4σ) (cm) | 0.04 ± 0.00 | 0.72 ± 0.19 | 0.05 ± 0.00 | 0.70 ± 0.34 |
| Exposure duration (s) | 120 | 120 | 120 | 120 |
| Radiant exposure (J/cm^2^) | 5,469.7 ± 55.6 | 2.4 ± 1.4 | 2,021.7 ± 7.3 | 2.4 ± 1.7 |
| Radiant energy (J) | 6.12 ± 0.07 | 0.84 ± 0.4 | 3.66 ± 0.01 | 0.74 ± 0.65 |

**Supplementary Table 1.** Profilometry of 660 and 810nm laser sources at scalp and cortical surfaces. Lasers used were 660nm MDL-III-660-FC-800 mW and 810nm MDL-III-810-FC-800 mW, (both from CNI Co Ltd, Changchun, China) coupled to a custom fibre patch cable (FG550UEC, Thor Labs, USA). D4σ given as average of x and y diameters.
